# Analysis of PTH serum concentration from internal jugular veins of patients with primary hyperparathyroidism^☆^^☆☆^

**DOI:** 10.1016/j.bjorl.2025.101605

**Published:** 2025-04-10

**Authors:** Davi Knoll Ribeiro, Murilo Catafestas das Neves, Rodrigo de Oliveira Santos, Marcio Abrahao

**Affiliations:** Universidade Federal de São Paulo (UNIFESP), Escola Paulista de Medicina, Departamento de Otorrinolaringologia e Cirurgia de Cabeça e Pescoço, São Paulo, SP, Brazil

**Keywords:** Rimary hyperparathyroidism, Parathyroidectomy, Parathyroid hormone, Jugular veins

## Abstract

**Objective:**

To evaluate whether the values of Parathyroid Hormone (PTH) collected from the internal jugular veins of patients with primary hyperparathyroidism can assist in the surgical approach.

**Methods:**

Prospective study of patients who underwent parathyroid adenoma excision by PHPT, collected right and left internal jugular vein blood sample for analysis of Parathyroid Hormone.

**Results:**

Twenty-nine patients underwent surgery. All patients had a decrease in peripheral PTH greater than 50% with a mean of 73.47%. PTH collection from the internal jugular veins was positive regarding the confirmation of parathyroid adenoma laterality in 22 cases (75.86%) and failure in 7 cases (24.14%), (p-value = 0.001). Comparing the success rates of the Methoxyisobutylisonitrile parathyroid scintigraphy (MIBI) tests, parathyroid Ultrasonography (USG) and PTH of internal jugulars in relation to location of adenoma laterality, we observed MIBI as localizer in 89.65% of the cases followed by the Jugular PTH with 75.86% and USG with 44.82%.

**Conclusion:**

PTH collection from the internal jugular veins is useful in patients with primary hyperparathyroidism who underwent surgery as a possible method of localization exams, indicating adenoma laterality.

**Leve of Evidence:**

Level III.

## Highlights

• Localization of parathyroid in primary disease sometimes is a challenge to surgeons.
• There are some methods available like scintigraphy with MIB and parathyroid ultrasound, but occasionally parathyroid would not be located.
• The blood collection of internal jugular veins and dosage of PTH can be useful as a method for the localization of parathyroid in patients with primary disease.
• The selective PTH collection from the jugular vein identified adenoma laterally in 75.86% of patients with primary hyperparathyroidism.

## Introduction

Primary Hyperparathyroidism (PHPT) is a common disease, ranking third among endocrinological disorders,^1^ affecting 1 in 700 patients,^2^ about 3% in postmenopausal women^3^ and 0.73% in men.^4^ HPTP is caused by a solitary adenoma in 80%–85% of patients.^5^

Minimally invasive parathyroidectomy is the surgical treatment of choice using preoperative imaging and intraoperative PTH.^6^, ^7^, ^8^ Patients whose parathyroid cannot be located by scintigraphy (MIBI) or parathyroid Ultrasound (USG) should undergo conventional exploration of the four glands.^9^, ^10^

PTH collection from the right and left internal jugular veins via femoral vein catheterization has been shown to be a safe and useful method in preoperative investigation and provides additional information for parathyroid adenoma localization when MIBI or USG are negative.^11^

Given this scenario, this study aimed at evaluating whether the collection of PTH from the internal jugular veins bilaterally helps to identify parathyroid adenoma laterality in patients with PHPT.

## Methods

This study was approved by the Research Ethics Committee, with the protocol number 1305.0014.11/2018. This is a prospective study, from April 2018 to April 2019, of all patients who underwent HPTP parathyroidectomy in a tertiary hospital by the Head and Neck Surgery team.

Data were collected from medical records on gender, age, localizatory exams, surgery performed and complications. The surgical technique and PTH collection were performed according to the usual technique previously published.^12^

As a study protocol, after cervical incision and prethyroid musculature dissection, both internal jugular veins were dissected for PTH collection under direct vision. At this time no exploration for identification of the parathyroid glands has been performed. After PTH collection, exploration was guided by localizatory exams. When USG and MIBI were concordant in identifying adenoma laterality, we opted for focal surgery and when discordant, bilateral conventional surgery.

Inclusion criteria were patients diagnosed with PHPT (elevated PTH and symptomatic or asymptomatic hypercalcemia) and eligible for surgery, who agreed to participate in the study by signing the consent form. Exclusion criteria included patients with suspicion or diagnosis of multiglandular disease.

For statistical analysis, numerical variables were expressed as mean and standard deviation of minimum and maximum values, and categorical variables as number and percentage. For nonparametric values, in comparisons at the same time, the Analysis of Variance (ANOVA) was used, followed by Tukey’s a posteriori test considering a statistically significant *p*-value <0.05.^13^, ^14^

## Results

The sample consisted of 29 patients, 23 women and 6 men, with a mean age of 62.9 years (between 31 and 84 years), submitted to focal surgeries in 13 cases and conventional surgeries in 16 cases according to the localizatory imaging exams.

The mean left jugular vein PTH was higher than the contralateral mean, and the right-side adenoma was found in 14 cases (48.27%) and on the left side in 15 cases (51.72%), represented in Table 1. We observed no relationship between adenoma size and the total value of peripheral PTH or internal jugular veins.

**Table 1:** Representation of mean PTH value and parathyroid adenoma location.

Variables	Right side	Left side
Mean PTH	69.46 pg/mL	542.9 pg/mL
Superior	7	11
Inferior	7	4

We obtained a peripheral PTH drop greater than 50% in all cases,^12^ and an average of 73.47% in the study.

All 29 patients underwent preoperative MIBI and US. In assessing the success in identifying the adenoma side between imaging exams and jugular PTH collection, we observed high jugular MIBI and PTH hit rates, with statistical significance (Table 2).

**Table 2:** Distribution of success in identifying the laterality of adenoma.

Success	N	%	p-value
MIBI	26	89.65%	<0.001^a^
Jugular PTH	22	75.86%	<0.001^a^
USG	13	44.82%	<0.505^a^

N, Number of Individuals; MIBI, Methoxy-Butyl Isonitrile parathyroid scintigraphy; PTH, Parathyroid Hormone; USG, Parathyroid Ultrasound.

a Determined by Mann-Whitney test.

By analyzing the absolute values of the jugular PTH, in the 22 successful cases in identifying adenoma laterality, maximum PTH concentration difference was 2798 pg/mL and minimum 17 pg/mL. In the 7 cases of failure, the maximum PTH concentration difference was 60 pg/mL and the minimum 2 pg/mL. In order to establish a jugular PTH cutoff value that indicates adenoma laterality, these values were transformed into percentages (Table 3).

**Table 3:** Representation of jugular PTH values difference (in percentage and absolute values) compared to the success in identifying adenoma laterality.

	Success		Failure	
Number of individuals	22		7	
Minimum jugular PTH difference	8.17%	17 pg/mL	0.70%	2 pg/mL
Maximum jugular PTH difference	93.26%	2798 pg/mL	15.70%	60 pg/mL

Having a maximum difference in jugular PTH of 15% in the failure group, values above this concentration could already be used as a reference for an estimate of probable adenoma laterality cutoff; however, considering the standard error of 10%, we estimated the cutoff value above 20%.

## Discussion

The use of PTH values of the internal jugular veins has proven to be an increasingly used tool for parathyroid surgeries, whether for patients with or without localizatory exams, but still with conflicting results regarding the reliability of the method.^10^, ^11^, ^15^ The present study demonstrated that there is a difference in the concentration of PTH values of the internal jugular veins in relation to adenoma laterality in patients with PHPT, in addition to obtaining a possible cutoff value in the concentration difference between the jugular veins. When the difference in concentration between the jugular PTH was greater than 20%, the side with the highest concentration was according to the location of the adenoma in 100% of the cases. We found no studies comparing PTH values of single-puncture jugular, only studies of selective jugular PTH collection through common femoral catheterization without comparing the concentration of the values, but with excellent results in the localization of adenomas.^16^

Applying the cutoff value of 20% concentration difference between the jugular PTH values, considering the standard error of 10% because we do not have enough patients for the sample calculation, in cases of bilateral cervical scanning where preoperative imaging exams were negative or conflicting (16 patients in our sample), using this method as a possible examination for localization of adenoma laterality, we could identify and avoid bilateral cervical exploration in 10 cases (62.5%). It is noteworthy that in a scenario where we would have negative or conflicting preoperative imaging examinations in our service, the average time to obtain the results of PTH values to perform the surgical approach is approximately 30 min. Thus, the ideal would be to perform the PTH collection of the jugular cases on an outpatient basis guided by USG so as not to spend surgical time waiting for this result. We need further work with negative and/or conflicting preoperative examinations with internal jugular PTH collections to establish this relationship and use it as a method for unilateral neck approach.

Maceri et al.^15^ observed a significant difference between the internal jugular vein PTH levels corresponding to the parathyroid adenoma side and that the greater the difference between the absolute jugular vein PTH values, the more accurate the adenoma laterality is defined. In our study, the jugular PTH value was positive in 75.86% of the cases, in addition to the fact that there is a difference between the right and left internal jugular vein PTH values that were unrelated to imaging or adenoma laterality. In 14 cases the adenoma was on the right side (48.27%) and on the left side in 15 cases (51.27%), and the mean concentration of PTH was 542.9 pg/mL and 69.46 pg/mL on the left and right side, respectively. No arguments were found in the literature to justify a difference between PTH values of the right-to-left internal jugular vein. It is possible that the venous drainage on the left side is slower than the contralateral side, thus presenting a higher PTH concentration on this side, but this hypothesis needs confirmation. Only one study by Wafae et al.^17^ was performed on the anatomy of the cervical drainage and its surgical application describe that the superior thyroid vein was identified in all dissected corpses, whereas the average vein corroborated values above in only 43% of the cases. Regarding the inferior thyroids vein, there is a series of anatomic variations however not justifying the above-corroborated values.

Ito et al.^10^ showed that PTH of the internal jugular veins compared to MIBI was 79.6% vs. 71.79% respectively; Alvarado et al.^18^ showed 76% vs. 36%, in our study compared to parathyroid USG, higher rates were observed in MIBI, followed by jugular PTH and USG respectively, 89.65% vs. 75.86% vs. 44.82% showing possible utility of this preoperative examination.

According to Ibraheem et al.,^11^ PTH collection from the internal jugular veins as a highly sensitive method (74%) should not be routinely used for preoperative localization of the parathyroid adenoma, but to guide surgical planning in cases of recurrent or persistent HPTP, when the localizatory imaging exams are negative. Ito et al.,^10^ however, along with Barczynski et al.^19^ showed that PTH of the internal jugular veins is a simple, safe and effective method and is an additional tool for adenoma localization in cases of negative MIBI and multiglandular diseases. The present study aimed only at analyzing the reliability of the jugular PTH collection, not related to the fact that the patients had persistent disease or non-localizatory preoperative imaging exams.

Our study is limited by the lack of a control group for comparative analysis and a moderate number of patients. Thus, research with a control group and a larger number of cases is necessary for future investigation and correlations so that PTH collection from the internal jugular veins becomes routine as a method of localizatory examination of patients with PPHT and surgical parathyroidectomy programming

## Conclusion

Selective PTH collection from the internal jugular veins identified adenoma laterality in 75.86% in patients with PTPH submitted to parathyroidectomy, establishing it as a possible method of identification of the adenoma and minimizing the indication of bilateral conventional surgeries in specific cases.

## Declaration of competing interest

The authors declare no conflicts of interest.
